# Derivation and validation of a new visceral adiposity index for predicting visceral obesity and cardiometabolic risk in a Korean population

**DOI:** 10.1371/journal.pone.0203787

**Published:** 2018-09-13

**Authors:** Sung-Kwan Oh, A-Ra Cho, Yu-Jin Kwon, Hye-Sun Lee, Ji-Won Lee

**Affiliations:** 1 Department of Family Medicine, Gangnam Severance Hospital, Yonsei University College of Medicine, Seoul, Republic of Korea; 2 Department of Family Medicine, Yong-In Severance Hospital, Yonsei University College of Medicine, Yong-In, Republic of Korea; 3 Biostatistics Collaboration Unit, Department of Research Affairs, Yonsei University College of Medicine, Seoul, Republic of Korea; International University of Health and Welfare, School of Medicine, JAPAN

## Abstract

**Objectives:**

The visceral adiposity index (VAI), an indirect marker of visceral adipose tissue, serves as a model associated with cardiometabolic risk, but has limitations regarding the Asian population. We sought to develop a new VAI (NVAI) for the Korean population and compare it to VAI for prediction of atherosclerotic cardiovascular disease (ASCVD) risk and development of major cardiovascular diseases (CVD) and stroke.

**Methods:**

Patients (969) who underwent visceral fat area measurement were analyzed. After exclusion, 539 patients (142 men, 397 women) were randomly divided into internal (n = 374) and external validation (n = 165) data set. The NVAI was developed using univariate and multivariate logistic regression with backward selection of predictors. Receiver operating characteristic (ROC) curve analysis and comparison of the area under the curve (AUC) verified the better predictor of ASCVD risk score. Additionally, nationwide population-based cross-sectional survey data (Korean National Health and Nutrition Examination Survey [KNHANES] 2008–2010, n = 29,235) was used to validate the NVAI’s ability to predict ASCVD risk and major CVD and stroke.

**Results:**

The NVAI better reflected visceral fat area in internal and external data sets, with AUCs of 0.911 (95% confidence interval [CI]: 0.882–0.940) and 0.879 (95% CI: 0.828–0.931), respectively. NVAI better discriminated for ASCVD risk (AUC = 0.892, 95% CI: 0.846–0.938) compared to VAI (0.559, 95% CI: 0.439–0.679). The NVAI also better predicted MI or angina, and stroke with AUCs of 0.771 (95% CI: 0.752–0.789), and 0.812 (95% CI: 0.794–0.830), respectively, compared with waist circumference (WC), body mass index (BMI), TG to HDL ratio, and VAI via KNHANES, in a statistically significant manner.

**Conclusions:**

The NVAI has advantages as a predictor of visceral obesity and is significantly associated with ASCVD risks and development of major CVD and stroke in the Korean population. The NVAI could be a screening tool for improved risk estimation related to visceral obesity.

## Introduction

It is well known that abdominal visceral fat is related to insulin resistance regardless of body mass index (BMI). In most studies, abdominal visceral fat is a better determinant of cardiometabolic risk factors such as hypertension, type 2 diabetes, and dyslipidemia than abdominal subcutaneous fat [1–8]. Furthermore, abdominal visceral fat has a stronger association with a risk of cardiovascular [3, 5, 8] and cerebrovascular disease compared with other anthropometric measures (e.g. BMI, total fat, and waist circumference [WC]) [9–11].

Computed tomography (CT) and magnetic resonance imaging (MRI) precisely and reliably quantify individual differences in abdominal fat distribution and distinguish visceral adipose tissue from subcutaneous adiposity, but both methods are expensive and CT has a high risk of radiation exposure [12]. Thus, Amato et al. established the visceral adiposity index (VAI) model that was based on a Caucasian population; this multivariate model includes non-invasive, simple parameters (WC, BMI, serum triglycerides [TG], and high-density lipoprotein [HDL] cholesterol levels) to incorporate functionality in deriving a measure of dysfunctional adipose tissue that is not directly visceral adiposity [13]. VAI significantly correlates with metabolic syndrome and cardiovascular risk [13]. However, there are conflicting data [14–17], partly due to the study of different populations and ethnicity groups [17–21]. The application of the VAI to non-Caucasian populations is limited, and there is little data regarding the Korean population.

Our study sought to develop a new VAI (NVAI) applicable to the Korean population and compare the efficacy of the NVAI for prediction of ASCVD risk and development of major cardiovascular diseases (CVD) and stroke.

## Methods

### Study population

**Fig 1:**
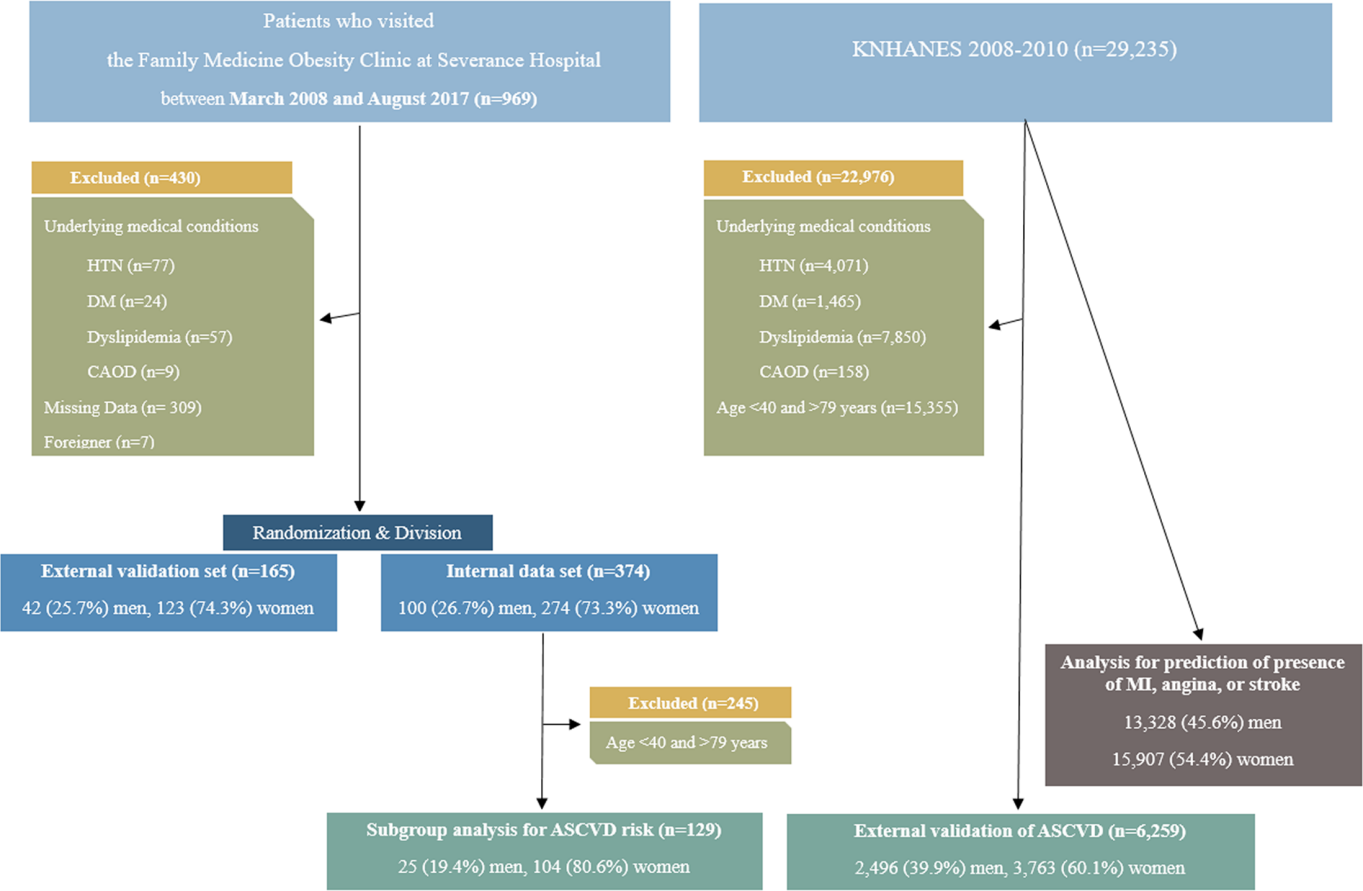
Flowchart for patient group selection. KNHANES, Korean National Health, and Nutrition Examination Survey; HTN, hypertension; DM, diabetes mellitus; CAOD, coronary artery occlusive disease; MI, myocardial infarction; ASCVD, atherosclerotic cardiovascular disease.

We analyzed 969 patients (262 men and 707 women) who voluntarily visited the Family medicine obesity clinic at the Severance Hospital from March 2008-May 2017 and received an abdominal CT scan for an obesity-related health check-up. Exclusion criteria were: a history of hypertension, diabetes, dyslipidemia, or coronary artery occlusive disease (CAOD). Missing data regarding metabolic parameters as well as foreigners were also excluded. After these exclusions, 539 patients were included for final analysis. In order to validate the NVAI, the 539 patients were randomly divided into an internal dataset (n = 374 [69.4% of total patients, 100 men and 274 women]) and an external validation set (n = 165 [30.6% of total patients, 42 men and 123 women]). This was a conventional statistical way to split the data according to a 7:3 proportion [22]. NVAI was developed using an internal data set and verified using an external data set. Additionally, we used national representative population-based cross-sectional survey data (Korean National Health and Nutrition Examination Survey [KNHANES] 2008–2010) to validate the predictive ability of the NVAI for the ASCVD risk and development of major CVD and stroke. After exclusion, 6,259 patients (aged 40–79 years; 2,496 men and 3,763 women) were enrolled and analyzed with regard to age, sex, race, diabetes status, smoking status, treated and untreated high blood pressure, systolic blood pressure, total serum cholesterol, and serum HDL-cholesterol to obtain the ASCVD 10-year risk score (Fig 1). A total of 29,235 patients (13,328 men and 15,907 women) were used to verify the predictive ability of the NVAI for major CVD and stroke (Fig 1). We retrospectively reviewed the medical records of participants. The medical record data was recorded by the investigator in such a way that the patients cannot be identified (i.e., by the investigator or others) either directly, or indirectly via linkage codes assigned to the data. Therefore, the need for consent was waived and the study was conducted in accordance with the Declaration of Helsinki and approved by the Institutional Review Board of Gangnam Severance Hospital (IRB No: 3-2018-0054).

### Clinical and anthropometric evaluation

All participants completed a uniform questionnaire detailing their medical history, medication usage, smoking status, and alcohol consumption (S1 Appendix). Height was measured to the closest 0.1 cm and the weight was measured to the closest 0.1 kg using an automatic height-weight scale (BSM 330, Biospace, Seoul, Korea). BMI was estimated based on weight (kg) divided by height (m^2^). WC was measured at the umbilicus with the patient in the standing position. The same person acquired all measurements of the anthropometric parameters throughout the study in order to reduce variations. Blood pressure was measured using an electronic manometer (BPBio320, Biospace) after more than 10 minutes of rest in a seated position, and the mean blood pressure (MBP) was calculated as [1/3 (systolic blood pressure) + 2/3 (diastolic blood pressure)] based on an average of two measurements.

Abdominal fat areas were calculated by CT (Tomoscan 350; Philips, Mahwah, NJ, USA). As described in detail previously [23], with the patient in a supine position, a 10-mm CT slice scan was acquired at the L4 to L5 level to measure the total abdominal and visceral fat area. The visceral fat area was quantified by delineating the intra-abdominal cavity at the internal aspect of the abdominal and oblique muscle walls surrounding the cavity and the posterior aspect of the vertebral body. The subcutaneous fat area was calculated by subtracting the visceral fat area from the total abdominal fat area. The coefficients of variation for inter- and intra-observer reproducibility were 1.4% and 0.5%, respectively.

With regard to smoking habits, a cigarette smoker was defined as a person who currently smokes and had smoked more than 100 cigarettes during their lifetime. The ingestion of alcohol was defined as more than two to three drinks per week.

### Biochemical analysis

Blood samples were obtained from an antecubital vein of each patient after an 8-hour overnight fast. Fasting serum glucose, total cholesterol, TG, and HDL-cholesterol were measured with the ADVIA 1650 Clinical Chemistry System (Siemens Medical Solutions, Tarrytown, NY, USA). Low-density lipoprotein (LDL) cholesterol was calculated using the Friedewald equation if the serum TG level was below 400 mg/dL. Fasting insulin was determined with an electrochemiluminescence immunoassay using an Elecsys 2010 instrument (Roche, Indianapolis, IN, USA).

### Definition of metabolic syndrome, visceral adiposity index, and ASCVD risk score

Metabolic syndrome was defined as meeting three or more of the following criteria based on the revised National Cholesterol Education Program Adult Treatment Panel III definition and the Korean Society for the Study of Obesity [24]: (1) abdominal obesity (WC ≥90 cm in men or ≥85 cm in women); (2) fasting TG of ≥150 mg/dL; (3) low HDL-cholesterol (<40 mg/dL in men or <50 mg/dL in women); (4) increased blood pressure (systolic blood pressure ≥130 mmHg, diastolic blood pressure ≥85 mmHg, or taking of anti-hypertensive medications); and (5) impaired fasting glucose (fasting glucose ≥100 mg/dL or use of insulin or hypoglycemic medication), as described previously in detail [25].

Visceral obesity was defined as a person whose visceral fat area was more than 100 cm^2^, as measured by CT [26].

VAI was defined based on WC, BMI, serum HDL-cholesterol, and TG levels, as described by Amato et al [13].

Males:VAI=[WC/(39.68+1.88×BMI)]×(TG/1.03)×(1.31/HDL)

Females:VAI=[WC/(36.58+1.89×BMI)]×(TG/0.81)×(1.52/HDL)

A total of 374 patients (internal dataset) was used to build the NVAI. We considered variables significantly associated with the visceral fat area (>100 cm^2^) via univariate analysis. We checked multicollinearity of the variables and included age, BMI, WC, MBP, fasting plasma glucose, insulin, TG, HDL-cholesterol, LDL-cholesterol, and current smoking and drinking status to perform a logistic regression with backward (Wald) selection of predictors. We set the criterion for p-values at 0.05 to select an entry or a removal. After performing seven steps of logistic regression with backward selection of predictors, the final model of the NVAI was composed of age, WC, MBP, TG, and HDL-cholesterol and is described below. The β value of each variable is stated with the standard error below each equation.

Male:NVAI=1/[1+exp{‑(‑21.858+(0.099×age)+(0.10×WC)+(0.12×MBP)+(0.006×TG)+(‑0.077×HDL))}]

β:‑21.858(6.33),age:0.099(0.036),WC:0.10(0.041),MBP:0.122(0.042),TG:0.006(0.003),HDL:‑0.077(0.039)

Female:NVAI=1/[1+exp{‑(‑18.765+(0.058×age)+(0.14×WC)+(0.057×MBP)+(0.004×TG)+(‑0.057×HDL))}]

β:‑18.765(2.95),age:0.058(0.015),WC:0.14(0.023),MBP:0.057(0.020),TG:0.004(0.003),HDL:‑0.057(0.018)

The ASCVD risk score is defined as non-fatal myocardial infarction, coronary heart disease death, or stroke by 2013 ACC/AHA guideline. This is pooled cohort equations for the prediction of ASCVD 10-year risk among patients who have never experienced any of these events in the past. We set cutoff value of 7.5%, which is guideline of starting moderate or high intensity statin to reduce ASCVD risk in adults [27, 28].

### Statistical analysis

Data are expressed as mean ± standard deviation (SD) or frequency (percentage). Variables were tested for normality using the Kolmogorov-Smirnov test. Differences between groups with univariate analysis were detected by independent sample t-test and Mann Whitney U-test for continuous variables or a Chi-square test for categorical variables. Partial Pearson correlation was conducted between VAI, NVAI, and clinical variables. Univariate and multivariate logistic regression with backward (Wald) selection of predictors were used to build the NVAI. Receiver operating characteristic (ROC) curve analysis was performed to determine the better predictor of visceral fat area >100 cm^2^ by the area under the curve (AUC). Comparison of AUCs was performed using the DeLong method. The ROC curve analysis and comparison of AUCs were also used to compare the predictability of the ASCVD risk and development of major CVD and stroke between the NVAI and other risk factors.

We utilized nomograms to verify the eligibility of the NVAI with external validation data (using R package version 3.1.3). The Hosmer and Lemeshow goodness-of-fit test was used to assess the acceptability of the predictive models, as it determined how well the nomogram was calibrated, i.e., close approximation between the observed probability and the predicted probability demonstrated good calibration and confirmed the exportability of the NVAI. The standardized Pearson and deviance residual analysis were also used to assess the suitability of the predictive model (S2 Appendix). The nomogram is intuitive, easily calculates the predictive model of the visceral fat area and can be used in routine clinical practice (S3 Appendix).

Statistical analyses were performed with SPSS software (version 23.0; SPSS Inc., Chicago, IL, USA) and R package (version 3.1.3). P-values <0.05 were considered statistically significant.

## Results

The clinical characteristics of the patients are shown in Table 1. The total data set included 142 men and 397 women (mean age 36.0 ± 12.5 years). The internal data set was composed of 100 men and 274 women (mean age 36.4 ± 12.9 years), and the external validation data set included 42 men and 123 women (mean age 35.1 ± 11.6 years).

**Table 1:** Clinical characteristics of the study population.

	Total (n = 539)		Internal data set (n = 374)		External validation (n = 165)	
Factors	Male (n = 142)	Female (n = 397)	Male (n = 100)	Female (n = 274)	Male (n = 42)	Female (n = 123)
Age (years)	35.3 ± 13.1	36.2 ± 12.3	35.4 ± 13.1	36.7 ± 12.9	34.9 ± 13.3	35.1 ± 11.0
BMI (kg/m^2^)	31.2 ± 4.7	27.9 ± 5.0	31.6 ± 4.6	27.7 ± 4.9	30.0 ± 4.7	28.2 ± 5.2
WC (cm)	106.3 ± 15.6	92.2 ± 10.8	107.3 ± 17.0	91.8 ± 10.2	103.7 ± 11.5	93.0 ± 12.1
Visceral fat area (cm^2^)	135.0 ± 56.5	92.3 ± 44.4	137.5 ± 55.3	91.4 ± 41.0	129.0 ± 59.5	94.5 ± 51.2
Subcutaneous fat area (cm^2^)	304.1 ± 129.1	280.4 ± 129.6	311.4 ± 123.7	279.8 ± 129.0	286.8 ± 141.0	281.7 ± 131.4
Systolic BP (mmHg)	134.6 ± 15.7	121.2 ± 13.7	135.1 ± 16.8	121.3 ± 13.6	131.0 ± 12.1	121.1 ± 14.1
Diastolic BP (mmHg)	79.6 ± 10.7	72.0 ± 9.3	80.3 ± 11.0	72.0 ± 9.0	77.9 ± 9.7	72.0 ± 10.0
MBP (mmHg)	97.9 ± 11.6	88.5 ± 10.0	98.9 ± 12.3	88.5 ± 9,8	95.6 ± 9.7	88.4 ± 10.6
Fasting plasma glucose (mg/dL)	99.6 ± 18.1	93.0 ± 14.7	100.3 ± 20.0	91.8 ± 10.9	98.0 ± 12.3	95.8 ± 20.7
Insulin (μIU/mL)	19.3 ± 27.7	12.2 ± 14.1	20.7 ± 32.1	12.6 ± 15.9	16.1 ± 11.8	11.4 ± 8.8
Total cholesterol (mg/dL)	194.9 ± 38.1	190.0 ± 37.8	197.3 ± 36.8	189.2 ± 37.1	189.3 ± 41.1	191.9 ± 39.4
TG (mg/dL)	162.7 ± 92.1	109.3 ± 60.3	168.9 ± 103.0	111.3 ± 63.1	148.0 ± 56.8	104.8 ± 53.4
HDL-cholesterol (mg/dL)	42.5 ± 8.5	53.1 ± 12.1	42.4 ± 8.5	52.9 ± 11.7	42.8 ± 8.7	53.6 ± 12.9
LDL-cholesterol (mg/dL)	124.6 ± 35.9	117.3 ± 33.5	127.0 ± 34.8	116.5 ± 32.5	118.8 ± 38.0	119.0 ± 35.8
TG/HDL ratio	4.1 ± 2.7	2.3 ± 1.6	4.3 ± 3.1	2.3 ± 1.7	3.6 ± 1.7	2.2 ± 1.5
VAI	4.4 ± 4.0	3.5 ± 3.2	1.1 ± 0.12	1.0 ± 0.09	1.1 ± 0.05	1.0 ± 0.07
NVAI	0.73 ± 0.27	0.36 ± 0.31	0.77 ± 0.22	0.37 ± 0.31	0.62 ± 0.35	0.32 ± 0.31
Current smoker, N (%)	41 (28.9%)	21 (5.3%)	31 (31%)	18 (6.6%)	10 (23.8%)	3 (3.4%)
Alcohol drinker, N (%)	66 (46.5%)	51 (12.8%)	49 (49%)	32 (11.7%)	17 (40.5%)	19 (15.4%)

Values are presented as means ± standard deviation (SD) for continuous variables or number (percentage) for categorical variables.

VAI is a sex-specific mathematical index based on WC, BMI, TG, and HDL-cholesterol levels, indirectly expressing visceral adipose dysfunction associated with cardio metabolic risk in a Caucasian Sicilian population.

The NVAI was derived from multivariate logistic regression analysis, based on age, WC, HDL-cholesterol levels, TG, and MBP, expressed by the following equation

Male: NVAI = 1/[1+exp{-(-21.858+(0.099×age) + (0.10×WC) + (0.12×MBP) + (0.006×TG)+(-0.077×HDL)}; β: -21.858 (6.33), age: 0.099 (0.036), WC: 0.10 (0.041), MBP: 0.122 (0.042), TG: 0.006 (0.003), HDL: -0.077 (0.039)

Female: NVAI = 1/[1+exp{-(-18.765 + (0.058×age) + (0.14×WC) + (0.057×MBP) + (0.004×TG) + (-0.057×HDL)}]; β: -18.765 (2.95), age: 0.058 (0.015), WC: 0.14 (0.023), MBP: 0.057 (0.020), TG: 0.004 (0.003), HDL: -0.057 (0.018)

BMI, body mass index; WC, waist circumference; BP, blood pressure; MBP, mean blood pressure; TG, triglyceride; HDL, high-density lipoprotein; LDL, low-density lipoprotein; VAI, visceral adiposity index; NVAI, new visceral adiposity index

S4 Appendix shows the clinical characteristics of the subgroup analysis for ASCVD 10-year risk (internal data set and a subgroup of KNHANES) and major CVD and stroke for the full data of KNHANES. The internal data set subgroup was composed of 25 men and 104 women (mean age 50.4 ± 8.7 years). The mean systolic blood pressure, total cholesterol, and HDL-cholesterol were 127.8 ± 17.8 mmHg, 196.4 ± 40.5 mg/dL, and 51.7 ± 11.0 mg/dL, respectively. The external validation set from the KNHANES subgroup included 2,496 men and 3,763 women (mean age 53.9 ± 10.7 years) with a mean systolic blood pressure, total cholesterol, and HDL-cholesterol of 117.0 ± 16.7 mmHg, 188.2 ± 27.1 mg/dL, and 52.3 ± 9.2 mg/dL, respectively. The total patients from KNHANES included 13,328 men and 15,907 women (mean age 39.0 ± 22.6 years) with a mean systolic blood pressure, total cholesterol, and HDL-cholesterol of 115.5 ± 17.5 mmHg, 183.6 ± 36.5 mg/dL, and 48.3 ± 10.9 mg/dL, respectively.

As shown in Table 2, significant positive correlations were found between VAI and visceral fat area, fasting glucose, fasting insulin, TG, LDL-cholesterol, number of metabolic syndrome components, and ASCVD risk score, whereas significant negative correlation was found between VAI and HDL-cholesterol levels after adjusting for age, sex, and BMI. A similar trend was found between the NVAI and anthropometric and metabolic parameters. The NVAI was positively associated with the visceral fat area, subcutaneous abdominal fat area, MBP, TG, LDL-cholesterol, and number of metabolic syndrome components, whereas it was negatively associated with HDL-cholesterol levels after adjusting for age, sex, and BMI.

**Table 2:** Partial correlation between VAI, NVAI, and clinical variables.

	VAI^a^		NVAI^a^		VAI^b^		NVAI^b^	
Factors	r	p-value	r	p-value	r	p-value	r	p-value
BMI	0.25	<0.001	0.72	<0.001	-	-	-	-
WC (cm)	0.24	<0.001	0.65	<0.001	0.083	0.11	0.26	<0.001
Visceral fat area (cm^2^)	0.29	<0.001	0.57	<0.001	0.19	<0.001	0.31	<0.001
Subcutaneous fat area (cm^2^)	0.11	0.031	0.54	<0.001	-0.062	0.24	0.15	0.003
Visceral fat area (>100 cm^2^)	0.34	<0.001	0.64	<0.001	0.26	<0.001	0.50	<0.001
Systolic BP (mmHg)	0.12	0.018	0.50	<0.001	0.024	0.64	0.33	<0.001
Diastolic BP (mmHg)	0.10	0.057	0.46	<0.001	0.011	0.83	0.32	<0.001
MBP (mmHg)	0.12	0.023	0.52	<0.001	0.019	0.72	0.35	<0.001
Fasting plasma glucose (mg/dL)	0.20	<0.001	0.21	<0.001	0.14	0.009	0.014	0.79
Insulin, (μIU/mL)	0.22	<0.001	0.24	<0.001	0.16	0.002	0.06	0.30
TG (mg/dL)	0.93	<0.001	0.43	<0.001	0.92	<0.001	0.39	<0.001
HDL-cholesterol (mg/dL)	-0.57	<0.001	-0.57	<0.001	-0.53	<0.001	-0.53	<0.001
LDL-cholesterol (mg/dL)	0.17	0.001	0.22	<0.001	0.13	0.016	0.13	0.016
Metabolic syndrome*	0.55	<0.001	0.63	<0.001	0.51	<0.001	0.52	<0.001

^a^ Adjusted for age and sex

^b^ Adjusted for age, sex, and BMI

*Metabolic syndrome was defined as fulfilling the modified National Cholesterol Education Program Adult Treatment Panel III and the Korean Society for the Study of Obesity definitions.

VAI, visceral adiposity index; NVAI, new visceral adiposity index; BMI, body mass index; WC, waist circumference; BP, blood pressure, MBP, mean blood pressure; TG, triglyceride; HDL, high-density lipoprotein; LDL, low-density lipoprotein

**Fig 2:**
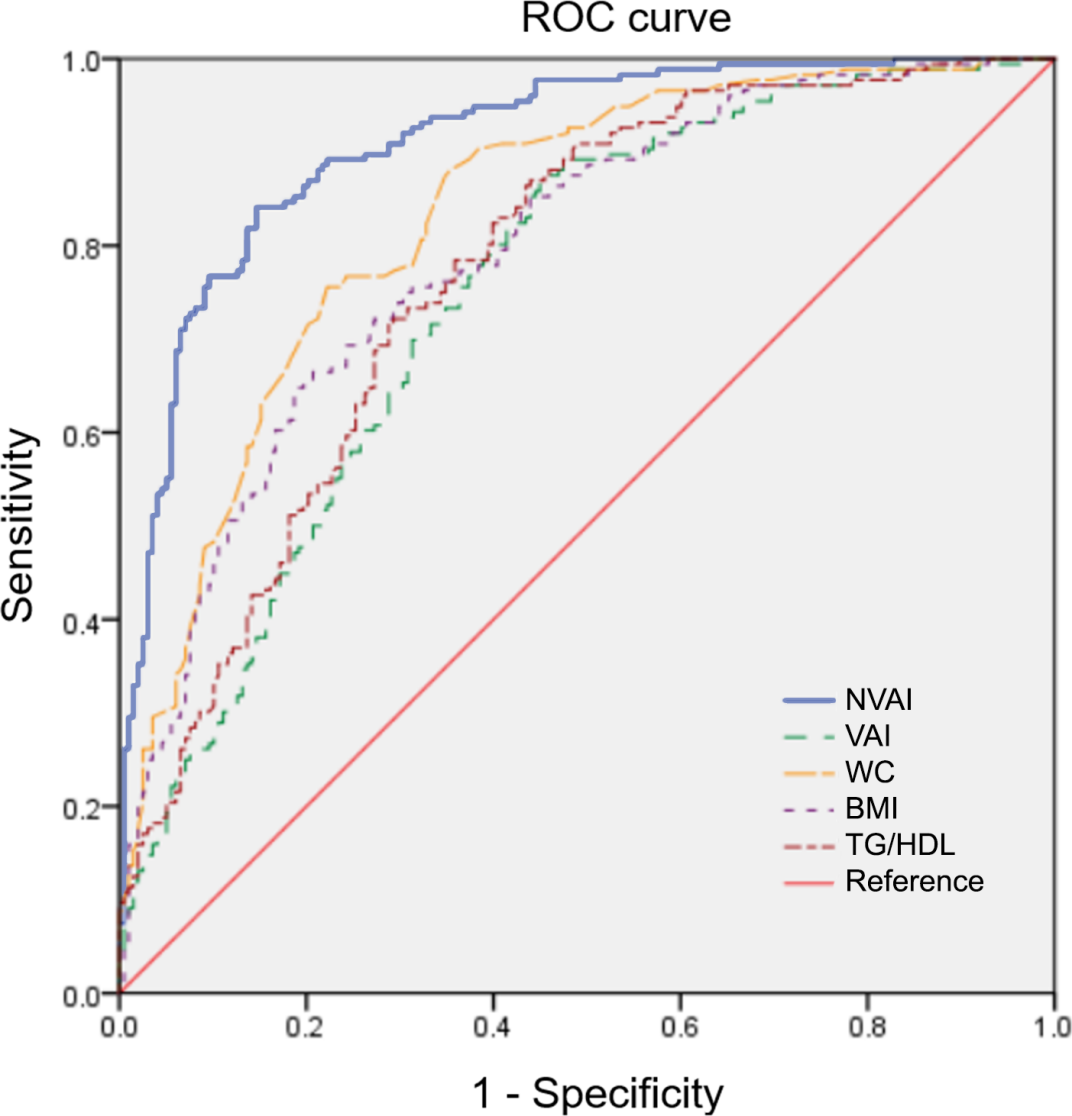
ROC curve for the prediction of visceral fat >100 cm^2^ using an internal data set. NVAI, new visceral adiposity index; VAI, visceral adiposity index; WC, waist circumference; BMI, body mass index; TG, triglyceride; HDL, high-density lipoprotein.

**Fig 3:**
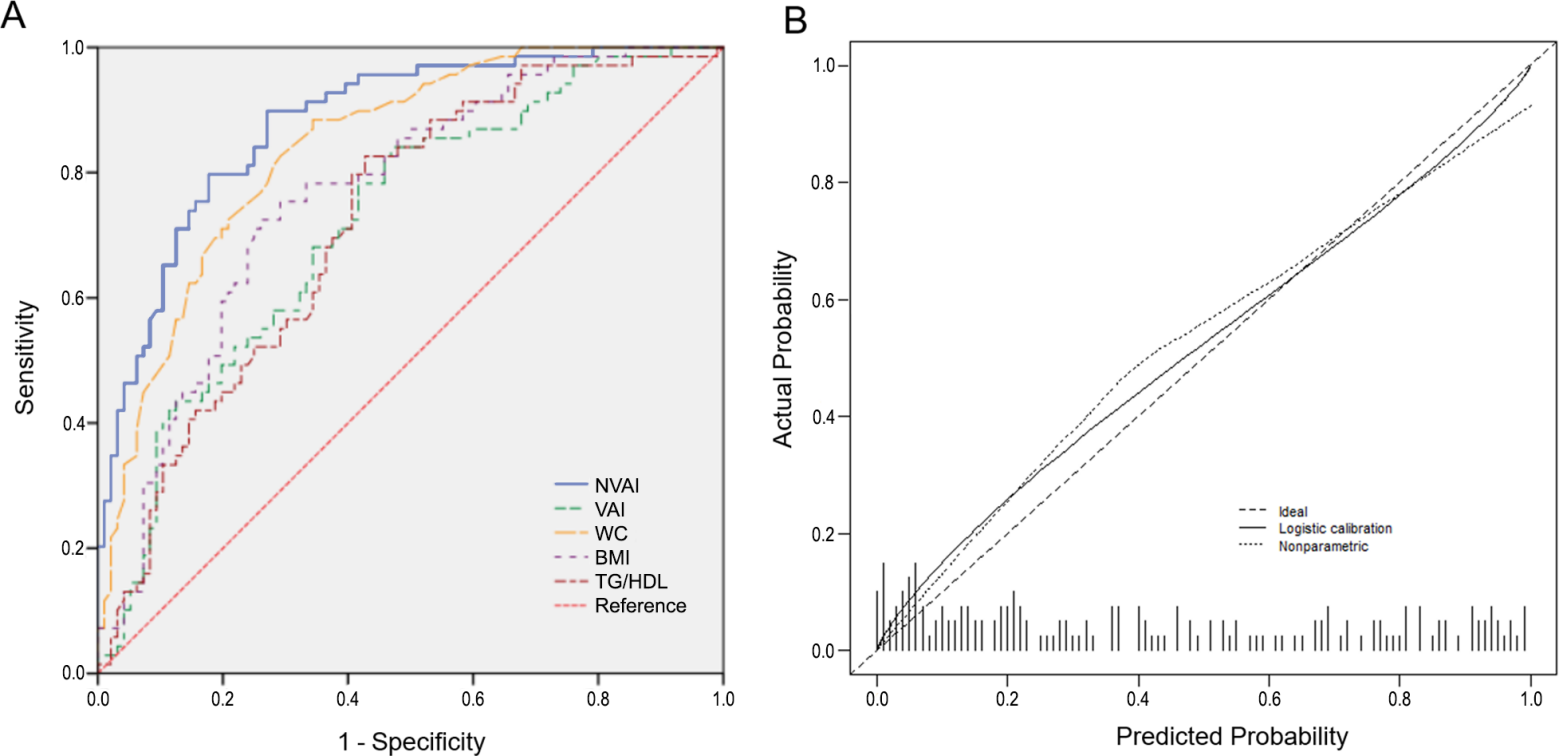
External validation of the NVAI using a validation set. (A) Discrimination of the NVAI was good, with a predicted visceral fat of >100 cm^2^, and values of 0.879 (SE: 0.026, 95% CI: 0.828–0.931) for the NVAI. (B) Calibrated plots demonstrate a close approximation to the logistic calibration of each nomogram, indicating good agreement between predicted and observed outcomes when using the NVAI. Value of intercept was 0.098, Value of slope was 0.834. WC, waist circumference; MBP, mean blood pressure; TG, triglyceride; HDL, high-density lipoprotein; ROC, receiver operating characteristic.

All AUCs of NVAI and associated variables were compared and found to be larger than 0.7. The predictive ability of the NVAI for visceral obesity displayed an AUC of 0.911 (SE: 0.015, 95% CI: 0.882–0.940) (Fig 2), which was significantly higher than WC, BMI, TG to HDL-cholesterol ratio, and the VAI (AUC = 0.751, SE: 0.025, 95% CI: 0.703–0.800). The VAI did not demonstrate a significant difference in prediction of visceral obesity compared with BMI or WC (Table 3). External validation of the NVAI for visceral obesity displayed an AUC of 0.879 (SE: 0.026, 95% CI: 0.828–0.931), which was higher than WC, BMI, TG to HDL-cholesterol, and the VAI (AUC = 0.720, SE: 0.040, 95% CI: 0.642–0.798), and calibration plots showed a close approximation to the logistic calibration of each nomogram (Fig 3).

**Table 3:** Post-hoc analysis of the AUC of NVAI for visceral obesity.

Index	AUC (SE, 95% CI)	Overall p-value	Post-hoc p-value*			
NVAI	0.911 (0.015, 0.882–0.940)	<0.001	Ref			
VAI	0.751 (0.025, 0.703–0.800)		<0.001	Ref		
WC	0.832 (0.021, 0.791–0.873)		<0.001	0.005	Ref	
BMI	0.812 (0.022, 0.769–0.855)		<0.001	0.04	0.08	Ref
TG/HDL ratio	0.789 (0.023, 0.744–0.834)		<0.001	0.03	0.05	0.3

*Post-hoc analyses were conducted using the DeLong method.

AUC, area under the curve; CI, confidence interval; NVAI, new visceral adiposity index; VAI, visceral adiposity index; WC, waist circumference; BMI, body mass index; TG, triglyceride; HDL, high-density lipoprotein

**Fig 4:**
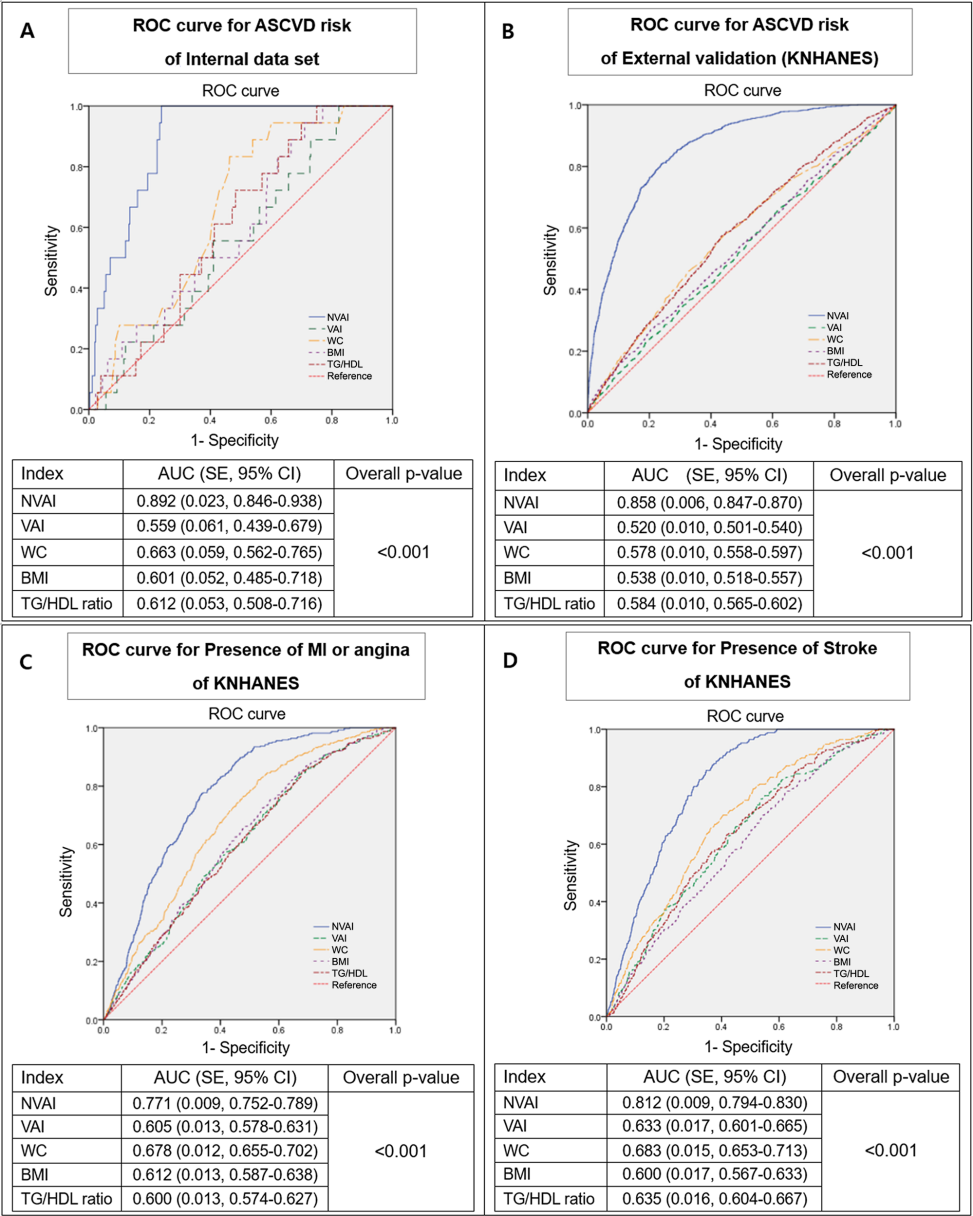
ROC curve and AUC comparison of the NVAI for ASCVD risk and CVD. (A) ROC curve for the prediction of the ASCVD risk using internal data set. (B) External validation for the prediction of the ASCVD risk using a subgroup of KNHANES IV-V. (C, D) ROC curve for predicting the presence of MI, angina, and stroke using the full data of KNHANES IV-V. Post-hoc analyses were conducted using the DeLong method. The cutoff value of the ASCVD 10-year risk score: ≥7.5%. ROC, receiver operating characteristic; ASCVD, atherosclerotic cardiovascular disease; NVAI, new visceral adiposity index; VAI, visceral adiposity index; WC, waist circumference; BMI, body mass index; TG, triglyceride; HDL, high-density lipoprotein; AUC, area under the curve; CI, confidence interval; KNHANES, Korean National Health and Nutrition Examination Survey; MI, myocardial infarction.

In order to determine the relative predictive ability of the NVAI for the ASCVD risk, ROC curve and comparison were also performed. The AUC was 0.892 (SE: 0.023, 95% CI: 0.846–0.938), which was significantly higher than WC, BMI, TG to HDL cholesterol ratio, and the VAI (AUC of 0.559, SE: 0.061, 95% CI: 0.439–0.679) (Fig 4A). External validation by KNHANES data was performed to validate these results, with an AUC of 0.858 (SE: 0.006, 95% CI: 0.847–0.870), a statistically significant difference compared to other factors relevant to the ASCVD risk (Fig 4B). When the predictive ability of the NVAI for a development of major CVD and stroke was tested using the full external validation data of KNHANES, the NVAI demonstrated statistically significant predictability for the presence of MI or angina with an AUC of 0.771 (SE: 0.009, 95% CI: 0.752–0.789) (Fig 4C). The presence of stroke was also statistically well-predicted by NVAI, with an AUC of 0.812 (SE: 0.009, 95% CI: 0.794–0.830) compared with WC, BMI, and the VAI (AUC of 0.633, SE: 0.017, 95% CI: 0.601–0.665) (Fig 4D).

## Discussion

CVD remains the leading cause of death worldwide, despite improved outcomes for CVD [29]. Recently, the declining trend in CVD has reached a plateau, necessitating an effort to generate more reliable data and powerful predictors to estimate the CVD burden and improve prevention and management [30].

Conflicting results have been reported with respect to the relationship of obesity as measured by BMI and the development of CVD [31], and the metabolic consequences of obesity vary according to the distribution of adipose tissue [3]. Indeed, visceral fat, not subcutaneous fat, is important for cardiometabolic complications [32]. The mechanisms behind the differential impacts of visceral and subcutaneous fat tissue on cardiometabolic risk include differences in adipocyte biology, inflammatory profiles, and connections to systemic circulation [32].

Although CT and MRI accurately distinguish between different types of body fat distribution, particularly visceral fat and subcutaneous fat tissue, these techniques are costly and not routinely available [33]. Therefore, there is a need for simple alternatives that can identify visceral adiposity, such as WC, but WC does not distinguish between subcutaneous and visceral fat mass [34]. Instead, the VAI was developed as an indirect marker of dysfunctional adipose tissue and incorporates both the presence of visceral adipose tissue and functional factors, which are indirectly expressed by high triglyceride/low HDL dyslipidemia [13]. In our study, we developed the NVAI, which is composed of age, WC, MBP, TG, and HDL-cholesterol in the Korean population. The NVAI significantly correlated with conventional cardiometabolic risk factors, and the AUC of NVAI for prediction of visceral obesity were significantly higher than those of the VAI. Next, we illustrated the usefulness of NVAI as a predictor of the ASCVD risk using an internal data set and nationwide population-based survey data (KNHANES). When we compared VAI and VAI components with NVAI regarding prediction of the ASCVD risk, the NVAI showed statistically significant prediction of the ASCVD risk in both the internal data set and KNHANES subgroup without hypertension, diabetes, dyslipidemia, and CAOD. In addition, NVAI had a better predictive value for major CVD and stroke with the full KNHANES population data.

The VAI and NVAI were generated by multivariate logistic regression, which was based on the same variables (WC, TG, and HDL-cholesterol) associated with abdominal visceral adiposity. However, NVAI additionally included age and MBP, while the VAI was determined from BMI. The exact reason for this difference is unknown, but ethnicity is considered the difference between the VAI (Caucasian population) and NVAI (Korean population). According to several studies, Asian populations tend to accumulate more visceral fat tissue at lower BMI and have a greater risk of metabolic complications and obesity-related CVD [3]. Therefore, just as BMI can incorrectly label muscular Asian people as overweight or obese, BMI can also erroneously categorize people with unhealthy amounts of fat as normal.

In our study, age was selected as an influential variable for visceral obesity. Cardiometabolic disease not only increases with age, but aging is also associated with the accumulation of visceral fat; this increase nearly quadruples between the ages of 25 and 65 years [35]. In addition, blood pressure was selected as a component of NVAI as well. There are several mechanisms postulated to explain the association of hypertension and obesity [36]. Hyperinsulinemia due to free fatty acid production from visceral adipose tissue could promote insulin resistance in the liver and skeletal muscle [37, 38], and hyperinsulinemia increases sympathetic activity and sodium tubular reabsorption [39]. Another assumption is that the compressive effect of visceral fat accumulation activates the renin angiotensin system, which increases sodium reabsorption, causing or exacerbating hypertension [40]. More longitudinal research is needed to determine the differences in the VAI indices according to race and ethnicity and how the differences affect ASCVD.

Our study has several limitations. First, this study was cross-sectional and limited in its ability to conclude causality. Second, ectopic fat is not measured, which may affect the relationship between the NVAI and ASCVD risk. Third, we could not calculate sample sizes initially; however, we divided enrolled patients into internal and external sets in a 7:3 proportion and we also used KNHANES data to verify the usefulness of NVAI. Fourth, this study was performed using a sample enriched for obesity from a single health-care center and is limited for generalized applications. Future longitudinal studies with larger data sets are needed to support our predictive model. Finally, the number of patients available for developing the NVAI is small, making it difficult to generalize and apply results to an entire population. However, NVAI was validated using nationally representative data to clarify the effects of NVAI on the ASCVD risk and development of major CVD and stroke.

In conclusion, we developed a reliable indicator for better prediction of visceral obesity than previous surrogate markers such as BMI, WC, and VAI in the Korean population. The NVAI has advantages as a predictor of visceral obesity itself and is significantly associated with the ASCVD risk and development of major CVD and stroke. In this regard, the NVAI could be used as a screening tool for improved risk estimation of major CVD or stroke.

## Supporting information

S1 Appendix Patient questionnaire of medical history. (PDF)

S2 Appendix Standardized pearson and deviance residual analysis of the NVAI. (TIF)

S3 Appendix Nomogram of the NVAI using clinical factors for predicting visceral obesity. (TIF)

S4 Appendix Clinical characteristics of the internal data set subgroup and the external validation set (subgroup of KNHANES) for the ASCVD 10-year risk (between 40 and 79 years of age) and total KNHANES for the presence of MI, angina, and stroke. (PDF)

## References

[1] Björntorp P. Visceral obesity: a “civilization syndrome”. Obesity. 1993;1(3):206–22. 10.1002/j.1550-8528.1993.tb00614.x 16350574

[2] Brunzell JD, Hokanson JE. Dyslipidemia of central obesity and insulin resistance. Diabetes care. 1999;22:C10 10189557

[3] Després J-P. Body fat distribution and risk of cardiovascular disease: an update. Circulation. 2012;126(10):1301–13. 10.1161/CIRCULATIONAHA.111.067264 22949540

[4] Després J-P, Lemieux I. Abdominal obesity and metabolic syndrome. Nature. 2006;444(7121):881 10.1038/nature05488 17167477

[5] Després J-P, Lemieux I, Bergeron J, Pibarot P, Mathieu P, Larose E, et al Abdominal obesity and the metabolic syndrome: contribution to global cardiometabolic risk. Arteriosclerosis, thrombosis, and vascular biology. 2008;28(6):1039–49. 10.1161/ATVBAHA.107.159228 18356555

[6] Jensen MD. Role of body fat distribution and the metabolic complications of obesity. The Journal of Clinical Endocrinology & Metabolism. 2008;93(11_supplement_1):s57–s63. 18987271 10.1210/jc.2008-1585 PMC2585758

[7] Pouliot M-C, Després J-P, Lemieux S, Moorjani S, Bouchard C, Tremblay A, et al Waist circumference and abdominal sagittal diameter: best simple anthropometric indexes of abdominal visceral adipose tissue accumulation and related cardiovascular risk in men and women. American journal of cardiology. 1994;73(7):460–8. 8141087 10.1016/0002-9149(94)90676-9

[8] Van Gaal LF, Mertens IL, Christophe E. Mechanisms linking obesity with cardiovascular disease. Nature. 2006;444(7121):875 10.1038/nature05487 17167476

[9] Rhéaume C, Leblanc M-È, Poirier P. Adiposity assessment: explaining the association between obesity, hypertension and stroke. Expert review of cardiovascular therapy. 2011;9(12):1557–64. 10.1586/erc.11.167 22103875

[10] Walker SP, Rimm EB, Ascherio A, Kawachi I, Stampfer MJ, Willett WC. Body size and fat distribution as predictors of stroke among US men. American Journal of Epidemiology. 1996;144(12):1143–50. 8956626 10.1093/oxfordjournals.aje.a008892

[11] Lu M, Ye W, ADAMI HO, Weiderpass E. Prospective study of body size and risk for stroke amongst women below age 60. Journal of internal medicine. 2006;260(5):442–50. 10.1111/j.1365-2796.2006.01706.x 17040250

[12] Graffy PM, Pickhardt PJ. Quantification of hepatic and visceral fat by CT and MR imaging: relevance to the obesity epidemic, metabolic syndrome and NAFLD. The British journal of radiology. 2016;89(1062):20151024 10.1259/bjr.20151024 26876880 PMC5258166

[13] Amato MC, Giordano C, Galia M, Criscimanna A, Vitabile S, Midiri M, et al Visceral Adiposity Index: a reliable indicator of visceral fat function associated with cardiometabolic risk. Diabetes care. 2010;33(4):920–2. 10.2337/dc09-1825 20067971 PMC2845052

[14] Oh JY, Sung YA, Lee HJ. The visceral adiposity index as a predictor of insulin resistance in young women with polycystic ovary syndrome. Obesity. 2013;21(8):1690–4. 10.1002/oby.20096 23585246

[15] Stępień M, Stępień A, Wlazeł RN, Paradowski M, Rizzo M, Banach M, et al Predictors of insulin resistance in patients with obesity: a pilot study. Angiology. 2014;65(1):22–30. 10.1177/0003319712468291 23267236

[16] Mohammadreza B, Farzad H, Davoud K. Prognostic significance of the complex" visceral adiposity index" vs. simple anthropometric measures: Tehran lipid and glucose study. Cardiovascular diabetology. 2012;11(1):20. 22394430 10.1186/1475-2840-11-20 PMC3376032

[17] Elisha B, Messier V, Karelis A, Coderre L, Bernard S, Prud’homme D, et al The visceral adiposity index: Relationship with cardiometabolic risk factors in obese and overweight postmenopausal women–A MONET group study. Applied Physiology, Nutrition, and Metabolism. 2013;38(8):892–9. 10.1139/apnm-2012-0307 23855278

[18] Després J-P, Couillard C, Gagnon J, Bergeron J, Leon AS, Rao D, et al Race, visceral adipose tissue, plasma lipids, and lipoprotein lipase activity in men and women: the Health, Risk Factors, Exercise Training, and Genetics (HERITAGE) family study. Arteriosclerosis, thrombosis, and vascular biology. 2000;20(8):1932–8. 10938014 10.1161/01.atv.20.8.1932

[19] U Lim TE, Buchthal SD, Latch M, Albright CL, Wilkens LR, Kolonel LN, Murphy SP, Chang L, Novotny R and Le Marchand L. Asian women have greater abdominal and visceral adiposity than Caucasian women with similar body mass index. Nutrition and Diabetes 2011;1. 10.1038/nutd.2011.2 PMC3302135 23449381

[20] Lear SA, Humphries KH, Kohli S, Chockalingam A, Frohlich JJ, Birmingham CL. Visceral adipose tissue accumulation differs according to ethnic background: results of the Multicultural Community Health Assessment Trial (M-CHAT)–. The American journal of clinical nutrition. 2007;86(2):353–9. 10.1093/ajcn/86.2.353 17684205

[21] Tanaka S, Horimai C, Katsukawa F. Ethnic differences in abdominal visceral fat accumulation between Japanese, African-Americans, and Caucasians: a meta-analysis. Acta diabetologica. 2003;40(1):s302–s4. 14618500 10.1007/s00592-003-0093-z

[22] James KE, White RF, Kraemer HC. Repeated split sample validation to assess logistic regression and recursive partitioning: an application to the prediction of cognitive impairment. Statistics in medicine. 2005;24(19):3019–35. 10.1002/sim.2154 16149128

[23] Lee JW, Im JA, Lee HR, Shim JY, Youn BS, Lee DC. Visceral adiposity is associated with serum retinol binding protein‐4 levels in healthy women. Obesity. 2007;15(9):2225–32. 10.1038/oby.2007.264 17890490

[24] Lee SY, Park HS, Kim DJ, Han JH, Kim SM, Cho GJ, et al Appropriate waist circumference cutoff points for central obesity in Korean adults. Diabetes research and clinical practice. 2007;75(1):72–80. 10.1016/j.diabres.2006.04.013 16735075

[25] Grundy SM, Cleeman JI, Daniels SR, Donato KA, Eckel RH, Franklin BA, et al Diagnosis and management of the metabolic syndrome: an American Heart Association/National Heart, Lung, and Blood Institute Scientific Statement. Circulation. 2005;112(17):2735–52. Epub 2005/09/15. 10.1161/CIRCULATIONAHA.105.169404 . 16157765

[26] Ryo M, Kishida K, Nakamura T, Yoshizumi T, Funahashi T, Shimomura I. Clinical significance of visceral adiposity assessed by computed tomography: a Japanese perspective. World journal of radiology. 2014;6(7):409 10.4329/wjr.v6.i7.409 25071881 PMC4109092

[27] Stone NJ, Robinson JG, Lichtenstein AH, Merz CNB, Blum CB, Eckel RH, et al 2013 ACC/AHA guideline on the treatment of blood cholesterol to reduce atherosclerotic cardiovascular risk in adults: a report of the American College of Cardiology/American Heart Association Task Force on Practice Guidelines. Journal of the American College of Cardiology. 2014;63(25 Part B):2889–934. 24239923 10.1016/j.jacc.2013.11.002

[28] Goff DC, Lloyd-Jones DM, Bennett G, Coady S, D’agostino RB, Gibbons R, et al 2013 ACC/AHA guideline on the assessment of cardiovascular risk: a report of the American College of Cardiology/American Heart Association Task Force on Practice Guidelines. Journal of the American College of Cardiology. 2014;63(25 Part B):2935–59. 24239921 10.1016/j.jacc.2013.11.005 PMC4700825

[29] Toth PP, Danese M, Villa G, Qian Y, Beaubrun A, Lira A, et al Estimated burden of cardiovascular disease and value-based price range for evolocumab in a high-risk, secondary-prevention population in the US payer context. Journal of medical economics. 2017;20(6):555–64. 10.1080/13696998.2017.1284078 28097904

[30] Benjamin EJ, Blaha MJ, Chiuve SE, Cushman M, Das SR, Deo R, et al Heart disease and stroke statistics-2017 update: a report from the American Heart Association. Circulation. 2017;135(10):e146–e603. 10.1161/CIR.0000000000000485 28122885 PMC5408160

[31] Hamer M, Stamatakis E. Metabolically healthy obesity and risk of all-cause and cardiovascular disease mortality. The Journal of Clinical Endocrinology & Metabolism. 2012;97(7):2482–8. 22508708 10.1210/jc.2011-3475 PMC3387408

[32] Sam S. Differential effect of subcutaneous abdominal and visceral adipose tissue on cardiometabolic risk. Hormone molecular biology and clinical investigation. 2018. 10.1515/hmbci-2018-0014 29522417

[33] Grauer WO, Moss AA, Cann CE, Goldberg HI. Quantification of body fat distribution in the abdomen using computed tomography. The American journal of clinical nutrition. 1984;39(4):631–7. 10.1093/ajcn/39.4.631 6711470

[34] Lee RK, Chung D, Chughtai B, Te AE, Kaplan SA. Central obesity as measured by waist circumference is predictive of severity of lower urinary tract symptoms. BJU international. 2012;110(4):540–5. 10.1111/j.1464-410X.2011.10819.x 22243806

[35] Hunter GR, Gower BA, Kane BL. Age related shift in visceral fat. International journal of body composition research. 2010;8(3):103 24834015 PMC4018766

[36] Faria AN, Ferreira SRG, Zanella MT. Impact of visceral fat on blood pressure and insulin sensitivity in hypertensive obese women. Obesity. 2002;10(12):1203–6. 10.1038/oby.2002.164 12490663

[37] Krotkiewski M, Björntorp P, Sjöström L, Smith U. Impact of obesity on metabolism in men and women. Importance of regional adipose tissue distribution. The Journal of clinical investigation. 1983;72(3):1150–62. 10.1172/JCI111040 6350364 PMC1129283

[38] Ferrannini E, Barrett E, Bevilacqua S, DeFronzo RA. Effect of fatty acids on glucose production and utilization in man. The Journal of clinical investigation. 1983;72(5):1737–47. 10.1172/JCI111133 6138367 PMC370462

[39] Moan A, Nordby G, Rostrup M, Eide I, Kjeldsen SE. Insulin sensitivity, sympathetic activity, and cardiovascular reactivity in young men. American journal of hypertension. 1995;8(3):268–75. 10.1016/0895-7061(94)00206-Q 7794576

[40] Mahabadi AA, Massaro JM, Rosito GA, Levy D, Murabito JM, Wolf PA, et al Association of pericardial fat, intrathoracic fat, and visceral abdominal fat with cardiovascular disease burden: the Framingham Heart Study. European heart journal. 2009;30(7):850–6. 10.1093/eurheartj/ehn573 19136488 PMC3693564

