# Supplementary material for: Derivation and validation of a new visceral adiposity index for predicting visceral obesity and cardiometabolic risk in a Korean population
Source: PLoS One. 2018 Sep 13;13(9):e0203787. doi: 10.1371/journal.pone.0203787 (PMC6136780; doi:10.1371/journal.pone.0203787)
Supplement: S1 Appendix — (PDF) [file pone.0203787.s001.pdf]

예진 기록지

검진 일자: ______________(연/월/일)

■ 이름:_______________ ■ 생년월일:___________________(연/월/일)

■ 키: (cm) ■ 몸무게: (kg) ■ 허리둘레: (cm) ■ 혈압: / (mmHg)

■ **현병력**

☐ 없음 ☐ 고혈압 ☐ 당뇨병 ☐ 결핵 ☐ 암 ☐고지혈증 ☐ 간염

☐ 갑상선질환 ☐ 심장질환 ☐ 뇌혈관질환 ☐ 기타( )

■ **약물 복용력**

☐ ____

■ **가족력**

☐ 있음 ( ) ☐ 없음

■ **음주력**

음주 ☐ 유 ☐무

술의 종류: ( ) 음주 횟수: (/주)

1회 음주량: ( ) 음주 시작 시기: ( )

끊은 시기: ( )

■ **흡연력**

흡연 ☐현재 흡연 ☐과거 흡연 ☐비흡연

하루의 흡연량: ( ) 흡연기간: ( )

흡연 시작시기: ( ) 끊은 시기: ( )

Patient Questionnaire

Date of health check-up: ____________________(yy/mm/dd)

■ Name: ______________________ ■ Date of Birth:________________________(yy/mm/dd)

■ Height: (cm) ■ Weight: (kg) ■ Waist circumference : (cm)

■ Blood pressure: / (mmHg)

■ **Present illness**

☐ None ☐ Hypertension ☐ Diabetes mellitus ☐ Tuberculosis ☐ Cancer

☐ Dyslipidemia ☐ Hepatitis ☐ Thyroid disease ☐ Cardiovascular disease

☐ Cerebrovascular disease ☐ Etc. ( )

■ **Medications in use**

____

■ **Family History**

☐ Yes ( ) ☐ None

■ **Drinking History**

Drinking status: ☐ Yes ☐ No

Alcohol type: ( ) Frequency: (/week)

Amount: ( ) Start year: ( )

Quit year: ( )

■ **Smoking History**

Smoking status: ☐ current smoker ☐ Ex-smoker ☐ Non-smoker

When did you start smoking? ( )

How much do you smoke a day? ( )

How long have you been smoking? ( )

*When did you quit smoking? ( ) * only Ex-smoker
