# Supplementary figures and images for: Derivation and validation of a new visceral adiposity index for predicting visceral obesity and cardiometabolic risk in a Korean population
Source: PLoS One. 2018 Sep 13;13(9):e0203787. doi: 10.1371/journal.pone.0203787 (PMC6136780; doi:10.1371/journal.pone.0203787)

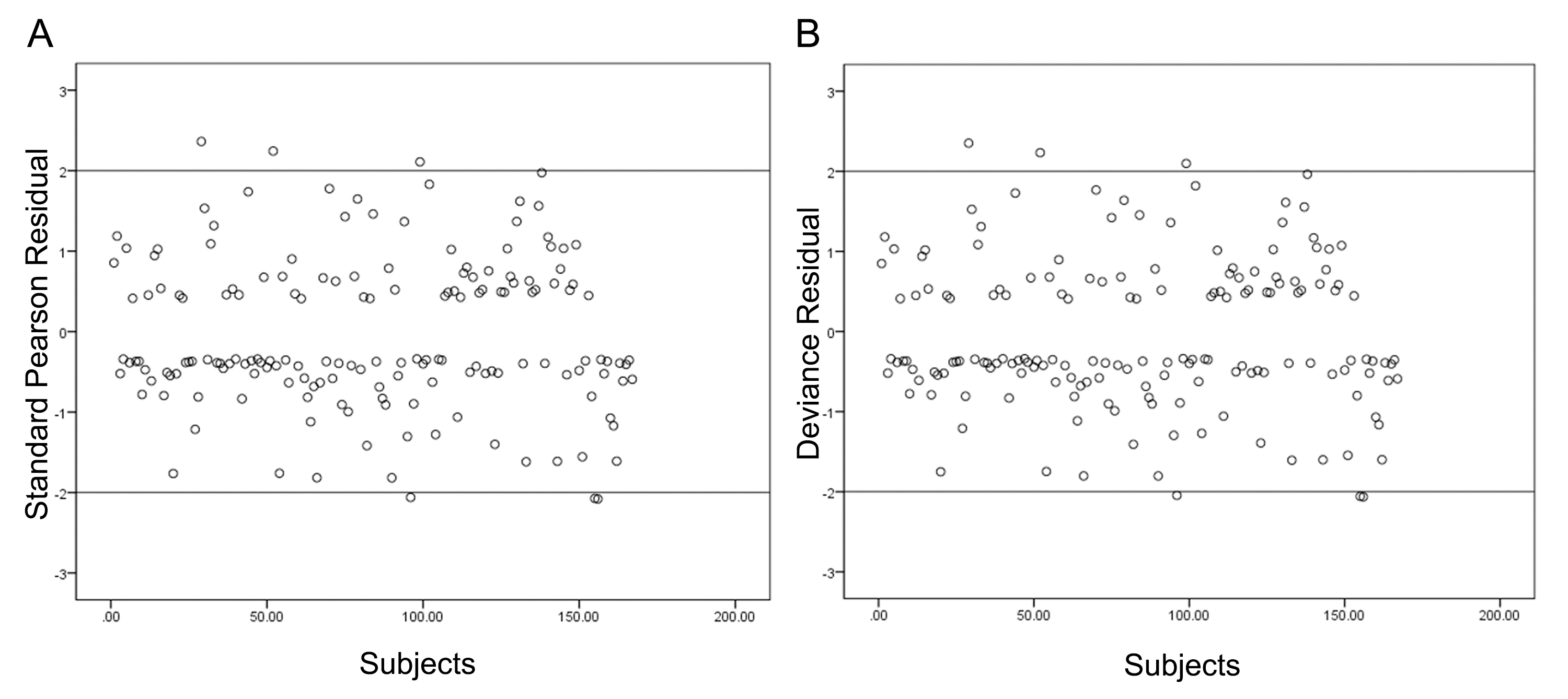

Supplement: S2 Appendix — (TIF) [file pone.0203787.s002.tif]

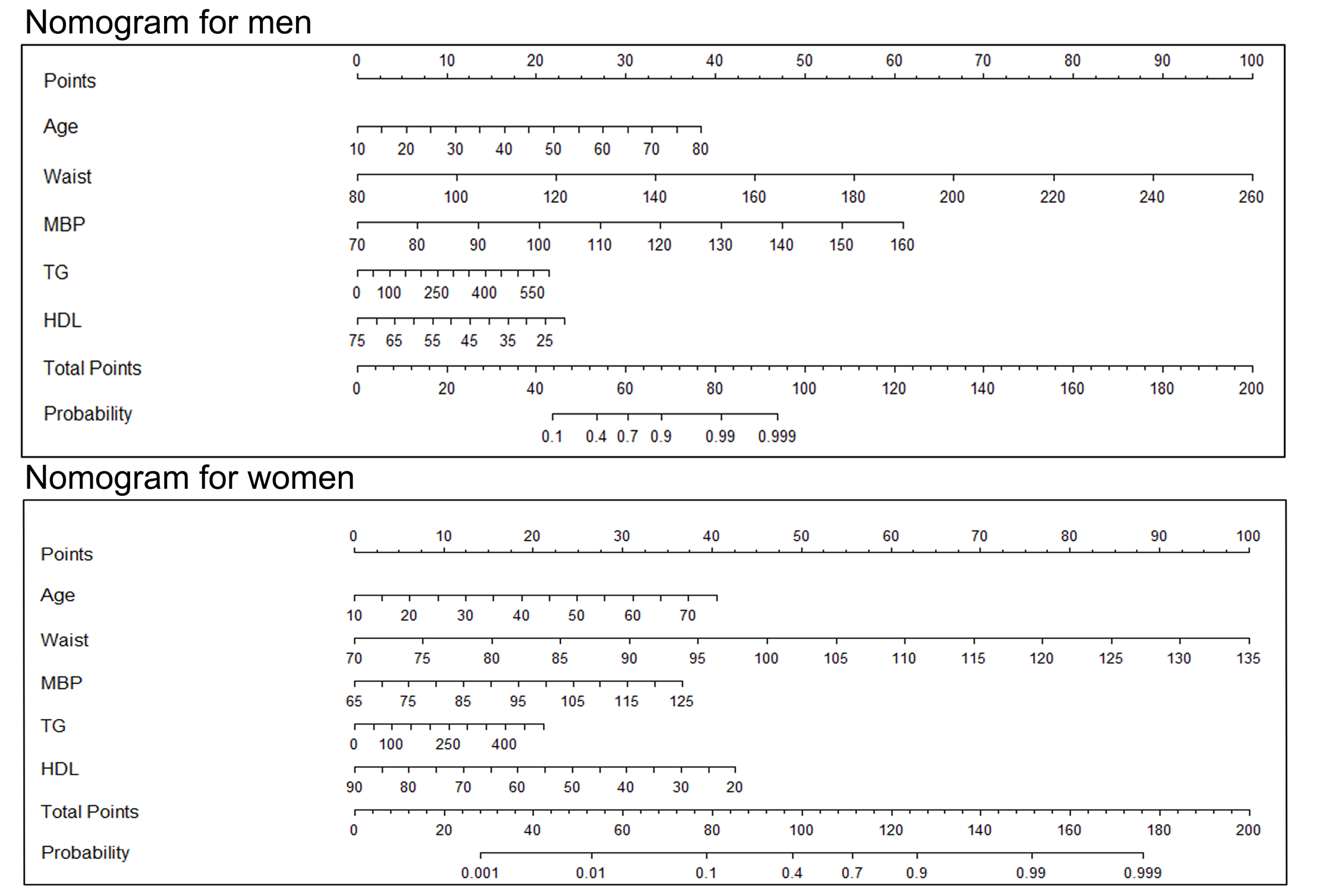

Supplement: S3 Appendix — (TIF) [file pone.0203787.s003.tif]
