# Supplementary material for: Derivation and validation of a new visceral adiposity index for predicting visceral obesity and cardiometabolic risk in a Korean population
Source: PLoS One. 2018 Sep 13;13(9):e0203787. doi: 10.1371/journal.pone.0203787 (PMC6136780; doi:10.1371/journal.pone.0203787)
Supplement: S4 Appendix — (PDF) [file pone.0203787.s004.pdf]

S4 Appendix. Clinical characteristics of the internal data set subgroup and the external validation set (subgroup of KNHANES) for the ASCVD 10-year risk (between 40 and 79 years of age) and total KNHANES for the presence of MI, angina, and stroke.

	Subgroup of internal data (n=129)		Subgroup of KNHANES (n=6,259)		Total KNHANES (n=29,235)	
**Factors**	**Men (n=25)**	**Women (n=104)**	**Men (n=2,496)**	**Women (n=3,763)**	**Men (n=13,328)**	**Women (n=15,907)**
Age (years)	51.1 ± 11.1	50.3 ± 8.1	54.9 ± 11.0	53.2 ± 10.4	37.4 ± 22.7	40.3 ± 22.5
BMI (kg/m^2)	31.1 ± 4.8	27.0 ± 4.6	23.1 ± 3.0	23.2 ± 3.0	22.4 ± 4.0	22.2 ± 4.1
WC (cm)	110.3 ± 29.5	91.3 ± 9.2	82.2 ± 8.6	78.0 ± 8.6	77.7 ± 14.5	74.3 ± 13.2
Systolic BP (mmHg)	142.8 ± 21.3	124.2 ± 14.8	119.5 ± 16.3	115.4 ± 16.7	117.1± 16.2	114.2 ± 18.3
MBP (mmHg)	106.0 ± 14.4	91.8 ± 9.6	91.4 ± 11.4	87.6 ± 11.4	89.0 ± 12.0	86.1 ± 12.3
Fasting plasma glucose (mg/dL)	104.4 ± 21.4	95.0 ± 11.4	96.7 ± 16.1	93.0 ± 11.9	98.4 ± 23.3	95.4 ± 21.5
Insulin (μIU/mL)	24.0 ± 51.7	10.2 ± 16.4	8.7 ± 5.1	9.2 ± 5.4	10.5 ± 6.3	10.6 ± 6.3
Total Cholesterol (mg/dL)	189.7 ± 33.8	198.0 ± 42.0	186.8 ± 27.6	189.0 ± 26.7	181.3 ± 36.6	185.4 ± 36.3
TG (mg/dL)	154.0 ± 71.4	117.0 ± 55.4	113.9 ± 69.7	93.2 ± 49.6	147.1 ± 130.3	112.3 ± 78.6
HDL cholesterol (mg/dL)	44.5 ± 8.6	53.4 ± 10.8	51.3 ± 9.5	53.0 ± 9.0	45.9 ± 10.3	50.3 ± 10.9
VAI	5.3 ± 3.3	4.7 ± 3.2	3.0 ± 2.3	3.4 ± 2.4	4.6 ± 5.3	4.6 ± 4.3
NVAI	0.92 ± 0.10	0.43 ± 0.32	0.53 ± 0.34	0.22 ± 0.24	0.47 ± 0.40	0.26 ± 0.32
Current smoker, N (%)	8 (32%)	4 (3.8%)	877 (35.1%)	168 (4.5%)	3,816 (28.6%)	690 (4.3%)

Current smoker is defined as a person who was currently smoking and had smoked over 100 cigarettes during their lifetime.

Values are presented as means ± standard deviation (SD) for continuous variables or number (percentage) for categorical variables.

The NVAI is derived from multivariate logistic regression analysis based on age, WC, HDL cholesterol levels, TG, and MBP and is expressed by the following equation:

Male: NVAI = 1/[1+exp{-(-21.858+(0.099×age)+(0.10×WC)+(0.12×MBP)+(0.006×TG)+(-0.077×HDL)}; β: -21.858 (6.33), age: 0.099 (0.036), WC: 0.10 (0.041), MBP: 0.122 (0.042), TG: 0.006 (0.003), HDL: -0.077 (0.039)

Female: NVAI = 1/[1+exp{-(-18.765+(0.058×age)+(0.14×WC)+(0.057×MBP)+(0.004×TG)+(-0.057×HDL)}]; β: -18.765 (2.95), age: 0.058 (0.015), WC: 0.14 (0.023), MBP: 0.057 (0.020), TG: 0.004 (0.003), HDL: -0.057 (0.018)

KNHANES, Korea National Health and Nutrition Examination Survey; ASCVD, Atherosclerotic cardiovascular disease; MI, myocardial infarction; BMI, body mass index; WC, waist circumference; BP, blood pressure; MBP, mean blood pressure; TG, triglyceride; HDL, high-density lipoprotein; VAI, visceral adiposity index; NVAI, new visceral adiposity index
